# The Association between Single Nucleotide Polymorphisms, including miR-499a Genetic Variants, and Dyslipidemia in Subjects Treated with Pharmacological or Phytochemical Lipid-Lowering Agents

**DOI:** 10.3390/ijms23105617

**Published:** 2022-05-17

**Authors:** Angelica Giuliani, Alberto Montesanto, Giulia Matacchione, Laura Graciotti, Deborah Ramini, Olga Protic, Roberta Galeazzi, Roberto Antonicelli, Elena Tortato, Anna Rita Bonfigli, Jacopo Sabbatinelli, Fabiola Olivieri

**Affiliations:** 1 Department of Clinical and Molecular Sciences, DISCLIMO, Università Politecnica delle Marche, 60126 Ancona, Italy; angelica.giuliani@staff.univpm.it (A.G.); g.matacchione@pm.univpm.it (G.M.); f.olivieri@univpm.it (F.O.); 2 Department of Biology, Ecology and Earth Sciences, University of Calabria, 87036 Rende, Italy; alberto.montesanto@unical.it; 3 Department of Excellence SBSP-Biomedical Sciences and Public Health, Università Politecnica delle Marche, 60126 Ancona, Italy; l.graciotti@staff.univpm.it; 4 Center of Clinical Pathology and Innovative Therapy, IRCCS INRCA, 60121 Ancona, Italy; d.ramini@pm.univpm.it; 5 Cardiology Unit, IRCCS INRCA, 60127 Ancona, Italy; o.protic@inrca.it (O.P.); r.antonicelli@inrca.it (R.A.); 6 Clinical Laboratory and Molecular Diagnostic, IRCCS INRCA, 60127 Ancona, Italy; r.galeazzi@inrca.it; 7 Metabolic Diseases and Diabetology Department, IRCCS INRCA, 60127 Ancona, Italy; e.tortato@inrca.it; 8 Scientific Direction, IRCCS INRCA, 60124 Ancona, Italy; a.bonfigli@inrca.it; 9 Laboratory Medicine Unit, Azienda Ospedaliero Universitaria Ospedali Riuniti, 60126 Ancona, Italy

**Keywords:** miR-499a, single nucleotide polymorphism, dyslipidemia, statins, cardiovascular risk, nutraceutical, blood lipids

## Abstract

Disorders of lipoprotein metabolism are among the major risk factors for cardiovascular disease (CVD) development. Single nucleotide polymorphisms (SNPs) have been associated with the individual variability in blood lipid profile and response to lipid-lowering treatments. Here, we genotyped 34 selected SNPs located in coding genes related to lipid metabolism, inflammation, coagulation, and a polymorphism in the *MIR499* gene—a microRNA previously linked to CVD—to evaluate the association with lipid trait in subjects with moderate dyslipidemia not on lipid-lowering treatment (Treatment-naïve (TN) cohort, *n* = 125) and in patients treated with statins (STAT cohort, *n* = 302). We also explored the association between SNPs and the effect of a novel phytochemical lipid-lowering treatment in the TN cohort. We found that 6 SNPs (in the *MIR499*, TNFA, CETP, SOD2, and VEGFA genes) were associated with lipid traits in the TN cohort, while no association was found with the response to twelve-week phytochemical treatment. In the STAT cohort, nine SNPs (in the *MIR499*, CETP, CYP2C9, IL6, ABCC2, PON1, IL10, and VEGFA genes) were associated with lipid traits, three of which were in common with the TN cohort. Interestingly, in both cohorts, the presence of the rs3746444 *MIR499* SNP was associated with a more favorable blood lipid profile. Our findings could add information to better understand the individual genetic variability in maintaining a low atherogenic lipid profile and the response to different lipid-lowering therapies.

## 1. Introduction

The cardiovascular disease (CVD) global burden has risen steadily over the past years [1]. High serum levels of total cholesterol (TC), low-density lipoprotein cholesterol (LDL-C), or triglycerides (TG)—i.e., dyslipidemia—are considered among the most prominent risk factors associated with CVD development [2]. Hypercholesterolemia is the most common form of dyslipidemia that can be caused by the inheritance (familial hypercholesterolemia, FH) of a major variant in specific coding genes, such as LDL receptor (LDL-R), apolipoprotein B (APOB) or E (APOE), and the Proprotein Convertase Subtilisin/Kexin type 9 (PCSK9) [3]. Nevertheless, an increased number of cases are diagnosed as polygenic hypercholesterolemia (PH) and may be attributed to the small additive effect of several single nucleotide variants located along the whole genome [4]. The huge technical improvements in genotyping allowed the identification of several genetic variants associated to CVDs and relevant risk factors. In recent years, in parallel to increased knowledge of the coding genome, new studies started to characterize disease-associated single nucleotide polymorphisms (SNPs) located within non-coding DNA regions [5]. We focused our analysis on a few SNPs located in selected coding genes previously related to lipid metabolism, inflammatory status, coagulation, drug metabolism, and on a polymorphism in the *MIR499* gene, a non-coding microRNA, to evaluate the association with the lipid trait in patients with moderate hypercholesterolemia not on lipid-lowering treatment and in patients treated with pharmacological (statins) and nutraceutical lipid-lowering treatments.

We selected miR-499a-5p since it was previously associated with lipid metabolism-related pathways [6,7,8,9,10], but evidence on this association is not conclusive [11]. The rs3746444 SNP in the *MIR499* gene influences the expression of miR-499a-5p and of its target genes, including OSBPL1A, which encodes for a member of the oxysterol-binding protein (OSBP) family, a group of intracellular lipid receptors [7]. Interestingly, significantly increased miR-499a-5p plasma levels were observed in patients affected by acute myocardial infarction (MI) [12,13,14,15], and rs3746444 polymorphism was previously identified as a marker of susceptibility to MI [16] and atrial fibrillation (AF) [8].

In this study, we analyzed 34 SNPs located in 22 selected coding genes and the rs3746444 polymorphism in the *MIR499* gene, in a total sample of 427 Caucasian patients affected by hypercholesterolemia, including subjects with moderate dyslipidemia and without any type of lipid-lowering therapy (*n* = 125) and the same subjects treated for 12 weeks with a new phytochemical product endowed with well-established anti-inflammatory properties [17,18] and potential lipid-lowering effects, and patients with dyslipidemia on statin treatment (*n* = 302).

The main aim of our study was to identify the association between selected SNPs and lipid traits in these different conditions.

## 2. Results

Clinical and metabolic assessments of 125 subjects affected by moderate dyslipidemia before phytochemical treatment (treatment-naïve cohort, TN) and 302 patients treated with lipid-lowering therapy (statin cohort, STAT) from at least one year are reported in Table 1. We selected 34 different SNPs located in genes related to lipid metabolism, inflammatory and coagulation status, and a SNP in the *MIR499* gene. Table 2 reports the complete list of the 34 selected SNPs, their position, and functional effects.

After the quality control (QC) phase of the 34 selected SNPs, we excluded three SNPs (rs1799837, rs366631, and rs72558195) that were monomorphic in both cohorts, and the rs2740574 SNP, which had a minor allele frequency (MAF) < 5% in both cohorts. The rs17238540 SNP, which showed a significant deviation from the HWE in the STAT cohort, was analyzed only in the TN cohort (see Supplementary Table S1). The final dataset included 30 high-quality SNPs that were tested for association with the baseline lipid trait in the TN cohort and 29 high-quality SNPs that were tested in the STAT cohort.

### 2.1. Associations of Selected SNPs with the Baseline Lipid Profile

**Figure 1:**
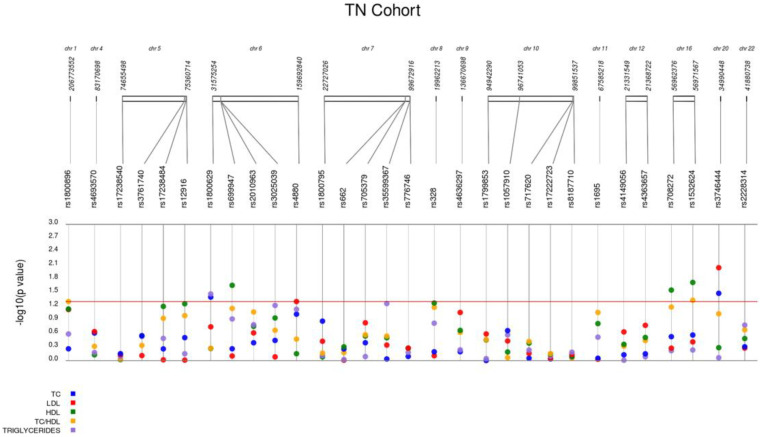
Results of the genetic associations with the baseline lipid traits in the treatment-naïve cohort.

Table 3 and Figure 1 summarize the results obtained from the association analyses in 125 subjects with moderate hypercholesterolemia not on lipid-lowering therapy (TN cohort).

**Figure 2:**
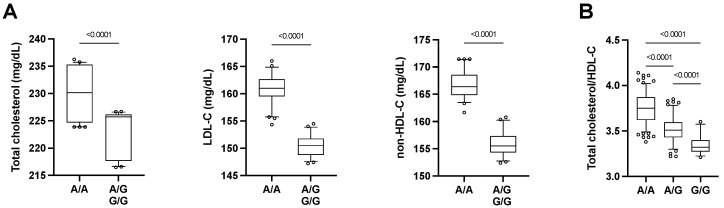
Blood lipid parameters, adjusted for age and gender, according to rs3746444 (MYH7B miR-499) genotypes in (**A**) 125 subjects with moderate dyslipidemia and not on lipid-lowering therapy and (A/A, *n* = 64; A/G + G/G, *n* = 52) (**B**) in 302 patients affected by dyslipidemia and treated with lipid-lowering therapy (A/A, *n* = 158; A/G, *n* = 116; G/G, *n* = 28). Whiskers of the boxplots mark the 5th and 95th percentiles, the boxes 25th percentile, median and 75th percentile, while extreme values are shown by circles. *p*-values for Tukey’s post-hoc comparisons following one-way ANCOVA.

After adjustment for age and gender, six SNPs were significantly associated with lipid trait in the TN cohort: two SNPs were associated with total cholesterol (TC) (rs1800629 and rs3746444), two with LDL-C (rs3746444 and rs4880), three with HDL-C (rs1532624, rs699947 and rs708272), one with triglycerides (rs1800629), one with the TC:HDL-C ratio (rs1532624), and one with non-HDL cholesterol (rs3746444). Some of them were associated with multiple traits (i.e., rs3746444, TC and LDL-C; rs1800629, TC and triglycerides; and rs1532624: HDL-C and TC:HDL-C ratio) (Table 3). Notably, subjects carrying the G allele for the rs3746444 SNP, which is located in the *MIR499* gene, had significantly lower levels of TC, LDL-C, and non-HDL-C (Figure 2A).

The six SNPs associated with blood lipid profile variables in the TN cohort were then analyzed as predictors of the response to the phytochemical product administered in a randomized clinical trial [19]. Results of the analysis, performed after adjustment for age and gender, revealed no significant interaction between phytochemical intervention and each SNP on variations in blood lipid parameters observed at the 12-week follow-up visit (Supplementary Table S2).

### 2.2. Associations of Selected SNPs with Lipid Profile in Subjects under Lipid-Lowering Therapy

The 29 high-quality SNPs were tested in 302 patients treated with statins from at least one year (STAT cohort). Results are summarized in Table 4.

After adjustment for age and gender, nine SNPs were significantly associated with blood lipid profile. Notably, three significant SNP associations, i.e., rs3746444, rs1532624, and rs708272, were common in the two cohorts. Interestingly, two of them were related to SNPs (rs1532624 and rs708272) located in the cholesteryl ester transfer protein (CETP) gene and were associated with HDL-C. Specifically, subjects homozygous for the A allele of either SNP had higher HDL-C compared to subjects carrying the major alleles. The rs3746444 *MIR499* SNP showed a significant association with the TC:HDL-C ratio (*p* = 0.016). Indeed, the TC:HDL-C ratio was lower in subjects carrying one (3.52 [0.09]) or two (3.35 [0.15]) G alleles compared to subjects with the A/A genotype (3.75 [0.09]) (Figure 2B). Two SNPs, i.e., rs1057910 (CYP2C9) and rs717620 (ABCC2), were associated with multiple lipid parameters (Table 4). In particular, subjects in the STAT cohort carrying the minor C allele for rs1057910 had higher TC, triglycerides, TC:HDL-C ratio, and non-HDL-cholesterol, while the minor T allele of the rs717620 SNP was associated with lower TC, triglycerides TC:HDL-C ratio, and non-HDL-cholesterol. Three SNPs related to systemic inflammation were associated with LDL-C (rs1800795, IL-6) and triglycerides (rs1800896, IL-10; rs2010963, VEGFA).

The association analysis of all analyzed SNPs with the European Society of Cardiology (ESC) CV risk categories highlighted significant gender-specific associations for rs3761740 (HMGCR) and rs705379 (PON1) in males. Specifically, subjects carrying the minor alleles (A and G, respectively) had a higher CV risk (*p* < 0.001 for rs3761740 and *p* = 0.022 for rs705379). Moreover, divergent gender-specific associations were observed for rs4149056 (SLCO). Indeed, in males the presence of the minor C allele was associated with lower CV risk (*p* = 0.044), while females carrying the minor allele had a significantly higher CV risk compared to subjects homozygous for the major T allele (*p* = 0.013). On the other hand, the rs3746444 (miR-499-5p) SNP was not significantly associated with CV risk.

### 2.3. Bioinformatic Prediction of miR-499a-5p Target Genes Related to Lipid Metabolism

It has been previously reported that the A→G SNP (rs3746444) affects the maturation of miR-499a-5p, but not its seed sequence, by converting a stable A-U base pair to a wobble G-U base pair in the miR-499 precursor (pre-miR-499), resulting in reduced levels of mature miR-499-5p [20]. The same report showed that the rs3746444 SNP negatively affected the suppression of miR-499-5p target mRNAs. Of note, miR-499a-3p, which is derived from the 3′ arm of pre-miR-499, has a considerably lower expression and is not affected by this SNP. Therefore, we conducted a comprehensive search of miR-499a-5p target genes related to lipid metabolism using the DIANA target prediction algorithm microT-CDS [21] and PathCards, an integrated database of human biological pathways and their annotations [22]. From the intersection between the two databases, we identified 23 miR-499a-5p target genes involved in the ‘Metabolism of lipids and lipoproteins’ pathway. Four miR-499a-5p target genes, i.e., FYN, LIPA, NPC1L1, and SH3KBP1, were found also in the ‘Lipoprotein metabolism’ Superpath, where they participate in pathways related to lipid digestion, mobilization, and transport and lipoprotein internalization and degradation (Supplementary Table S3).

## 3. Discussion

Here, we analyzed 30 selected SNPs located in 22 genes, including *MIR499*, in a total of 427 subjects affected by moderate dyslipidemia, with and without phytochemical lipid-lowering therapies and patients with hyperlipidemia treated with statin, to identify the association between SNPs, lipid profile, and different lipid-lowering treatments.

We found that six SNPs located in genes coding for TNFA (chr. 6), CETP (chr. 16), SOD2 (chr. 6), VEGFA (chr. 6), and MYH7B (miR-499, chr. 20), previously investigated in the framework of lipid metabolism, were significantly associated with lipid traits in the treatment-naïve (TN) cohort. However, no significant effect of these SNPs was found on the response to a twelve-week treatment with a phytochemical administered to these subjects as a potential lipid-lowering supplement in the context of a randomized clinical trial [19]. Here, we aimed at exploring whether interindividual genetic variations in genes involved in systemic inflammation and lipid metabolism affected the response to the polyphenols and plant sterols contained in the dietary supplements. Our negative results may be attributed to the formulation of the phytochemical, e.g., doses and proportions of the single compounds in the mixture, non-optimal bioavailability, and poor adherence in the dietary supplementation consumption.

Association between the TNFA rs1800629 SNP and blood lipid levels produced conflicting results so far, mainly due to a wide variability of ethnicity, baseline CV risk, and interventions [23,24,25]. Here, we showed that the minor A allele is associated with higher TC and triglycerides. In contrast with a previous report showing a weak association between the rs4880 SOD2 G allele and higher LDL-C in Arab individuals [26], we observed that subjects homozygous for the G variant have lower LDL-C. However, the association observed in our cohort was very weak and warrants further exploration. Although the relation between lipid profiles and VEGFA expression has not yet been clearly defined, several observations suggested a potential implication of VEGFA in lipid metabolism [27,28]. Our study is the first report showing a positive association between the VEGFA rs699947 A variant and increased HDL-C in Caucasian subjects. Interestingly, this association was lost in the statin (STAT) cohort in which, on the other hand, a positive association between the rs2010963 VEGFA SNP and triglycerides emerged. Our findings are in line with previous evidence showing that the G allele of the rs2010963 SNP, which was prevalent in our cohort, and the minor rs699947 A allele are associated with lower VEGF circulating levels [29]. Notably, increased VEGF serum levels have been linked to atherosclerotic plaque progression and a higher incidence of CV events [30,31].

Further, in the STAT cohort, significant associations between lipid profile and SPNs of genes involved in systemic inflammation have been found. Specifically, the rs1800795 IL-6 SNP was associated with lower levels of LDL-C, in agreement with a previous report [32]. Conversely, individuals carrying the C allele for the rs1800896 IL-10 SNP had higher serum triglycerides levels. The strongest association with blood lipids in the STAT cohort were obtained for the ABCC2 and CYP2C9 SNPs. Interestingly, the T variant of the rs717620 ABCC2 SNP was associated with a dose-decrease or a switch to another lipid-lowering drug in simvastatin users due to a dramatic reduction in cholesterol levels [33], a finding that was recapitulated in our sample. While the rs1057910 CYP2C9 SNP had been previously linked to a higher risk of myopathy in statin users [34], data on the association with blood lipids in statin users is lacking. Here, we observed higher TC, non-HDL-C, and triglyceride levels in subjects carrying the C allele of this SNP. The CYP2C9 variants are worth investigating since this member of the cytochrome P450 family has a major role in the metabolism of most statins and has more than 60 reported genetic variants [35]. Moreover, we observed in both cohorts an association between higher HDL-C and the minor alleles of rs1532624 and rs708272 SNPs of CETP, a protein involved in the transfer of cholesteryl esters from HDL-C to other lipoproteins [36], confirming the results of several studies performed on a multitude of different ethnicities [37,38].

So far, only very few genetic studies have attempted to identify SNPs in miRNA genes that could be associated with CVD and related risk factors, including dyslipidemia. The miR-499 rs3746444 SNP has been analyzed in a variety of human diseases, including cancer [39,40], myocardial infarction [20], and hypertension [6]. The rs3746444 SNP consists of an A to G nucleotide substitution that creates a mismatch in the stem loop of the miR-499 precursor, affecting the maturation of both miR-499a-5p and miR-499a-3p [41]. Interestingly, miR-499 is encoded by two genes—*MIR499A* and *MIR499B*—that are in the same intronic region of the *MYH7B* gene (chr. 19) and transcribed in opposite directions [42]. Whereas expression data are available for the sense direction transcribed miR-499a, it is currently unknown whether miR-499b is expressed. Notably, evidence showed that the G allele of the rs3746444 polymorphism largely decreased miR-499a levels and this SNP was associated with lipid trait [7]. The association between the rs3746444 SNP and blood lipids was confirmed by multiple reports showing reduced triglyceride levels in subjects with the GG genotype [7,10,11]. In our cohort of treatment-naïve subjects affected by moderate dyslipidemia, we observed that miR-499 rs3746444 GG genotypes were characterized by increased levels of HDL-C and reduced levels of TC, compared to AA genotype. This finding was partly mitigated in the STAT cohort, in which the G allele of the SNP was associated only with a reduced TC:HDL-C ratio. Therefore, our results confirm that subjects carrying the miR-499 rs3746444 G allele are characterized by a lower pro-atherogenic lipid profile.

We further evaluated whether the investigated SNPs could contribute to improve cardiovascular (CV) risk estimation. To this purpose, we analyzed the SNPs in the STAT cohort grouped according to the CV risk levels proposed by the ESC [43]. The association analysis highlighted significant gender-specific associations for rs3761740 (HMGCR) and rs705379 (paraoxonase 1, PON1) in males, and divergent associations for rs4149056 (SLCO). Several variants of HMGCR, i.e., the pharmacological target of statins, had been linked to overall CV risk [44], but no reports on the specific role of rs3761740 were available. Although the PON1 rs705379 minor allele was more prevalent among males with a higher CV risk, this variant was associated with a more pronounced LDL-C reduction following statin therapy. The lipid-modifying, antioxidant, and anti-inflammatory properties of PON1 have been implicated in the prevention of atherosclerosis and its complications [45]. Interestingly, a protective role of genetic variants of PON1 towards increased in LDL-C and CV risk has been previously highlighted [46]. A previous study had found an association between the presence of the minor allele of the rs4149056 (SLCO) variant and high levels of LDL-C, but not with the risk of CV events, probably due to gender-specific associations that were not previously investigated [47].

Finally, we searched to identify some potential target genes of miR-499a-5p (Supplementary Table S3). As a potential miR-499 target, we identified the gene LIPA, which encodes for the lysosomal acid lipase (LAL), an enzyme responsible for the hydrolysis of triglycerides and cholesteryl esters (CE), resulting in the release of unesterified cholesterol and free fatty acids [48]. Mutations of LAL have been associated with CE and TG accumulation in the liver, spleen, and macrophages, resulting in liver failure, accelerated atherosclerosis, and premature death [49]. OSBPL1A, a member of the intracellular lipid receptors, was previously identified as a specific target of miR-499a-5p, and a reduced OSBPL1A activity was associated with a more pro-atherogenic lipid trait characterized by low plasma HDL-C levels [7,50].

Our study has some limitations that need to be addressed, including the limited sample size and the lack of patient stratification according to type/dose of statin medication and pretreatment cholesterol levels. However, by considering only the high-quality SNPs, we believe that our sample could be considered as representative of a real-world population of elderly subjects.

In conclusion, our findings may pave the way to better understand the individual variability in maintaining a low atherogenic lipid profile and responding to different lipid-lowering therapies. Indeed, the study of the inter-individual genetic variability in genes involved in lipid metabolism could help to identify subgroups of subjects in which statins fail to induce a reduction in total and LDL cholesterol. To this purpose, genetic research into statin response might lead to a better understanding of pharmacokinetics and pharmacodynamics, thus improving their clinical application in terms of drug type and dose. Further studies exploring the effects of the individual genetic makeup on the response to lipid-lowering treatments in larger cohorts may contribute to developing more personalized preventive strategies.

## 4. Materials and Methods

### 4.1. Study Population

The analyzed sample included (i) 125 participants affected by moderate hypercholesterolemia, without any type of lipid-lowering therapy (treatment-naïve cohort, TN) and (ii) 302 hypercholesterolemic patients treated with statins (statin treatment cohort, STAT). The TN cohort was recruited based on the following criteria: age of 40 years or older, total blood cholesterol levels of 200-250 mg/dL and no history of clinically relevant cardiovascular events. Exclusion criteria included a therapy with statins or food supplements with lipid-reducing effects or with anticoagulants, hypothyroidism, or hyperthyroidism; diabetes; intestinal malabsorption, acute illnesses, neoplastic disease or life expectancy less than one year; statin non-related liver disease; severe chronic renal failure; participation in a clinical trial of intervention for lipid modulations in the previous three months; presence of cognitive disorders and other impediments that do not guarantee the correct adherence to the study treatments; current or presumed pregnancy and pregnancy planning and incapacity; and impossibility or unavailability to sign the written consent. Participants were enrolled for participation in a randomized, double-blind, placebo-controlled, parallel group clinical trial (ACTRN12619000170123) evaluating the lipid-lowering effect of a dietary supplement [19]. Briefly, subjects were randomized to receive two capsules a day—one capsule taken 15 min before lunch and one capsule 15 min before dinner—of either a placebo or a dietary supplement containing 400 mg phytosterols, 100 mg bergamot fruit, 20 mg olive fruit extract, and 52 mcg vitamin K2. This formulation provided a flavonoid content of 98 mg and a total polyphenol content of 19.6 mg. The study lasted 12 weeks and outcomes were assessed at 6 and 12 weeks post-intervention commencement.

The STAT cohort was recruited based on the following criteria: age of 40 years or older and therapy with statins for at least 1 year. Hypercholesterolemic patients were recruited at the Cardiology Unit of the IRCCS INRCA of Ancona, Italy. All enrolled patients were Caucasian. Peripheral blood samples for DNA extraction were collected in EDTA tubes upon receipt of informed consent from all patients. The study was conducted in accordance with the Declaration of Helsinki and approved by the Ethics Committee of IRCCS INRCA (protocol code INRCA 17018; date of approval, 21 December 2017).

### 4.2. Data Collection

Complete blood count, blood lipid profile including triglycerides, total cholesterol (TC), low-density lipoprotein cholesterol (LDL), high-density lipoprotein cholesterol (HDL), lipoprotein(a), and inflammatory markers such as *hs*-CRP and IL-6 were determined by standard methods. The ten-year risk of fatal cardiovascular disease was calculated for each patient in the STAT-cohort using the SCORE algorithm [51]. Patients were then categorized into four categories of CV risk (low-risk, moderate-risk, high-risk, very-high risk) according to the 2019 ESC/EAS Guidelines for the management of dyslipidemia [43].

### 4.3. DNA Extraction and Genotyping

DNA was extracted from whole blood with the Qiagen DNA extraction kit (Qiagen Co., Hilden, Germany) according to the manufacturer’s recommendations. Genotyping analysis was performed by using SEQUENOM MassArray iPLEX technology (Sequenom, San Diego, CA, USA), following the manufacturer’s instructions. Genotype calls were analyzed by using SEQUENOM Typer 4.0 software and the individual spectrograms were checked to evaluate the presence of calling errors.

### 4.4. Statistical Analysis and Quality Control of Genotyping Data

Demographic, clinical characteristics, and outcomes data were summarized with counts and percentages for categorical variables, means (standard deviations) for normally distributed continuous variables, and medians (with interquartile ranges) for other continuous variables. The Kolmogorov–Smirnov test was used to check the normality of the related variables.

After genotype calling, the dataset was subjected to a battery of QC tests. SNPs were excluded if (i) they were monomorphic; (ii) they had a minor allele frequency (MAF) < 5% in both cohorts; (iii) they had a significant deviation from Hardy–Weinberg equilibrium (HWE) (*p* < 0.05), and (iv) a missing frequency higher than 10%. For each SNP, allele and genotype frequencies were estimated by gene counting from the observed genotypes. HWE was tested by Fisher’s exact test. Additive genetic effects were assumed for each SNP, with a value of 0, 1, or 2 being assigned based on the number of minor allele copies. When the additive genetic model was no longer applicable due to low MAF, the codominant model was used. Variables related to the blood lipid profiles were compared according to the rs3746444 genotypes using a one-way ANCOVA, adjusted for age and gender, followed by Tukey’s post-hoc comparisons. Data were analyzed using R (version 4.1.0). *p* values < 0.05 were deemed as significant.

## Figures and Tables

**Table 1:** Clinical and metabolic variables of the enrolled subjects.

	Patients Affected by Moderate Dyslipidemia Not on Lipid-Lowering Therapy (*n* = 125)	Patients with Dyslipidemia Treated with Lipid-Lowering Therapy (*n* = 302)	*p*
Age (years)	58.0 (8.7)	69.3 (7.9)	<0.001
Sex (males, %)	53 (42%)	140 (46%)	0.455
BMI (Kg/m^2^)	25.6 (4.2)	27.1 (4.4)	0.001
Weight (Kg)	72.0 (14.8)	73.1 (14.3)	0.474
Glucose (mg/dL)	92.9 (9.3)	121.0 (44.3)	<0.001
Total-cholesterol (mg/dL)	228.14 (21.2)	194.9 (42.6)	<0.001
HDL-cholesterol (mg/dL)	65.3 (16.4)	56.5 (15.2)	<0.001
LDL-cholesterol (mg/dL)	157.2 (20.9)	110.3 (32.1)	<0.001
Total cholesterol/HDL	3.7 (0.9)	3.62 (1.0)	0.439
Triglycerides (mg/dL)	111.6 (52.5)	118.8 (59.2)	0.238
*hs*-CRP (mg/L)	0.2 (0.4)	3.1 (9.0)	<0.001
Creatine kinase (U/L)	114.4 (72.9)	123.4 (73.9)	0.251
Lp(a) (mg/dL)	276.4 (324.7)	269.0 (272.5)	0.810
Myoglobin (mg/dL)	34.7 (21.3)	43.5 (22.8)	<0.001
Monocytes (n/mm^3^)	0.4 (0.1)	0.4 (0.1)	0.999
Neutrophils (n/mm^3^)	3.3 (1.1)	3.6 (1.2)	0.016
Lymphocytes (n/mm^3^)	2.1 (0.6)	2.0 (0.5)	0.077
Creatinine (mg/dL)	0.9 (0.2)	0.9 (0.3)	0.999

Variables are expressed as mean (standard deviation). BMI, body mass index; *hs*-CRP, high-sensitive C-reactive protein; Lp(a), lipoprotein(a); HDL, high-density lipoproteins; LDL, low-density lipoproteins. *p*-values for two-tailed Student’s *t* test and for Chi-squared test.

**Table 2:** Summary of the 34 selected SNPs in 22 genes associated with inflammation and lipid metabolism.

SNP ID	Chr.	Position	Locus	Functional Implication	Alleles	Type
rs3746444	20	34,990,448	MYH7B MIR-499	-	A/G	Intronic MYH7B, miR-499
rs366631	1	109,709,850	GSTM5	DM	A	upstream_transcript_variant
rs1800896	1	206,773,552	IL-10	I/C	T/C	intron_variant
rs4693570	4	83,170,698	100 kb downstream of COQ2	LM	T/C	Intergenic variant
rs17238540	5	74,655,498	HMGCR	LM	T/G	intron_variant
rs3761740	5	75,336,308	HMGCR	LM	C/A	upstream_transcript_variant
rs17238484	5	75,352,671	HMGCR	LM	G/T	intron_variant
rs12916	5	75,360,714	HMGCR	LM	T/C	3_prime_UTR_variant
rs1800629	6	31,575,254	TNFA	I/C	G/A	upstream_transcript_variant
rs699947	6	43,768,652	VEGFA	I/C	C/A	upstream_transcript_variant
rs2010963	6	43,770,613	VEGFA	I/C	G/C	upstream_transcript_variant
rs3025039	6	43,784,799	VEGFA	I/C	C/T	3_prime_UTR_variant
rs4880	6	159,692,840	SOD2	I/C	A/G	missense_variant
rs1800795	7	22,727,026	IL-6	I/C	G/C	intron_variant
rs662	7	95,308,134	PON1	LM	T/C	missense_variant
rs705379	7	95,324,583	PON1	LM	A/G	upstream_transcript_variant
rs35599367	7	99,366,316	CYP3A4	DM	G/A	intron_variant
rs776746	7	99,672,916	CYP3A5	DM	C/T	intron_variant
rs2740574	7	99,784,473	CYP3A4	DM	T/C	upstream_transcript_variant
rs328	8	19,962,213	LPL	LM	C/G	stop_gained, coding_sequence_variant
rs4636297	9	136,670,698	EGFL7	I/C	G/A	downstream_transcript_variant
rs1799853	10	94,942,290	CYP2C9	DM	C/T	missense_variant
rs72558195	10	95,064,886	CYP2C8	DM	G/A	stop_gained, missense_variant
rs1057910	10	96,741,053	CYP2C9	DM	A/C	missense_variant
rs717620	10	99,782,821	ABCC2	DM	C/T	upstream_transcript_variant
rs17222723	10	99,836,239	ABCC2	DM	T/A	missense_variant
rs8187710	10	99,851,537	ABCC2	DM	G/A	missense_variant
rs1695	11	67,585,218	GSTP1	DM	A/G	missense_variant
rs1799837	11	116,837,537	APOA1	LM	C/T	5_prime_UTR_variant
rs4149056	12	21,331,549	SLCO1B1	DM	T/C	missense_variant
rs4363657	12	21,368,722	SLCO1B1	DM	T/C	intron_variant
rs708272	16	56,962,376	CETP	LM	G/A	intron_variant
rs1532624	16	56,971,567	CETP	LM	C/A	intron_variant
rs2228314	22	41,880,738	SREBF2	LM	G/C	missense_variant

ABCC2: ATP Binding Cassette Subfamily C Member 2; APOA1: apolipoprotein A1; CETP: cholesterol ester transfer protein; COQ2: Coenzyme Q2, Polyprenyltransferase; CYP: cytochrome P450; DM: drug metabolism; EGFL7: EGF Like Domain Multiple 7; GSTM5: Glutathione S-Transferase Mu 5; GSTP1: Glutathione S-Transferase Pi 1; HMGCR: 3-hydroxy-3-methyglutaryl-CoA reductase; I/C: inflammation and coagulation; IL: interleukin; LM: lipid metabolism; LPL: lipoprotein lipase; LTA: Lymphotoxin-α; MYH7B: myosin heavy chain 7B, intronic miR-499; PON1: paraoxonase 1; SOD2: superoxide dismutase 2; SLCO1B1: Solute Carrier Organic Anion Transporter Family Member 1B1; SREBF2: Sterol Regulatory Element Binding Transcription Factor 2; TNFA: tumor necrosis factor alpha; VEGF: vascular endothelial growth factor.

**Table 3:** Associations between polymorphisms and lipid profile in 125 subjects with moderate dyslipidemia not on lipid-lowering therapy (treatment-naïve cohort, TN).

						*p*-Values					
SNP ID	Gene	Alleles	MAF	HWE	Call Rate	TC	LDL-C	HDL-C	TRIG	TC:HDL-C Ratio	Non-HDL-C
rs3746444	*MYH7B* miR-499	A/G	24.1	0.210	92.80	**0.033 ↓**	**0.009 ↓**	0.519	0.863	0.088	**0.009 ↓**
rs1800629	TNFA	G/A	10.3	1.000	93.60	**0.040 ↑**	0.180	0.541	**0.034 ↑**	0.530	0.110
rs708272	CETP	G/A	40.0	1.000	92.00	0.297	0.532	**0.028 ↑**	0.604	0.068	0.570
rs1532624	CETP	C/A	41.5	0.707	93.60	0.273	0.387	**0.019 ↑**	0.584	**0.049 ↓**	0.540
rs4880	SOD2	A/G	46.6	1.000	93.60	0.095	**0.049 ↓**	0.704	0.074	0.340	0.051
rs699947	VEGFA	C/A	38.5	0.845	93.60	0.548	0.790	**0.022 ↑**	0.121	0.064	0.280

*p*-values for log-additive model. Significant associations are in bold. HDL, high-density lipoprotein; HWE, Hardy-Weinberg equilibrium; LDL, low-density lipoprotein; MAF, minor allele frequencies; TC, total cholesterol. ↓/↑ indicate significantly lower or higher levels of the variable in subjects carrying the minor allele.

**Table 4:** Association analyses between polymorphisms and lipid profile in 302 subjects with dyslipidemia on statin lipid-lowering therapy (STAT cohort).

						*p*-Values					
SNP ID	Gene	Alleles	MAF	HWE	Call Rate	TC	LDL-C	HDL-C	TRIG	TC:HDL-C Ratio	Non-HDL-C
rs3746444	*MYH7B* miR-499a	A/G	28.5	0.32	100	0.850	0.970	0.079	0.310	**0.016 ↓**	0.380
rs1532624	CETP	C/A	40.8	0.63	99.34	0.490	0.220	**0.035 ↑**	0.089	0.160	0.930
rs708272	CETP	G/A	39.1	0.72	100	0.540	0.260	**0.049 ↑**	0.100	0.170	0.910
rs1057910	CYP2C9	A/C	8.1	0.71	100	**0.020 ↑**	0.790	0.990	**0.016 ↑**	**0.028 ↑**	**0.015 ↑**
rs1800795	IL-6	G/C	28.2	0.89	99.67	0.340	**0.020 ↓**	0.190	0.510	0.190	0.130
rs717620	ABCC2	C/T	16.6	0.41	100	**0.049 ↓**	**0.0082 ↓**	0.210	0.230	**0.016 ↓**	**0.011 ↓**
rs705379	PON1	A/G	46.8	0.35	99.01	0.150	**0.024 ↓**	0.860	1.000	0.580	0.160
rs1800896	IL-10	T/C	37.3	0.33	100	0.790	0.990	0.270	**0.0011 ↑**	0.093	0.480
rs2010963	VEGFA	G/C	38.4	0.9	100	0.670	0.960	0.190	**0.043 ↑**	0.160	0.340

*p*-values for log-additive model. Significant associations are in bold. HDL, high-density lipoprotein; HWE, Hardy-Weinberg equilibrium; LDL, low-density lipoprotein; MAF, minor allele frequencies; TC, total cholesterol. ↓/↑ indicate significantly lower or higher levels of the variable in subjects carrying the minor allele.

## Data Availability

The data presented in this study are available on request from the corresponding author.

## Supplementary Materials

The following supporting information can be downloaded at: https://www.mdpi.com/article/10.3390/ijms23105617/s1.
