# Supplementary material for: The Association between Single Nucleotide Polymorphisms, including miR-499a Genetic Variants, and Dyslipidemia in Subjects Treated with Pharmacological or Phytochemical Lipid-Lowering Agents
Source: Int J Mol Sci. 2022 May 17;23(10):5617. doi: 10.3390/ijms23105617 (PMC9145435; doi:10.3390/ijms23105617)
Supplement: Supplementary file 1 [file ijms-23-05617-s001.zip › ijms-1718821-supplementary.pdf]

Supplementary Table S1. Summary of the 34 selected SNPs in 22 genes associated with inflammation and lipid metabolism. For each cohort, the minor allele frequency (MAF), the p-value for the Hardy-Weinberg equilibrium (HWE), and the percentage of missing genotypes are reported.

						TN cohort			STAT cohort		
Selected	SNP	CHR	Position	Locus	Allele	MAF	HWE (P-value)	Missing (%)	MAF	HWE (P-value)	Missing (%)
yes	rs1057910	10	96741053	CYP2C9	A/C	7.7	1.00	6.4	8.1	0.705	0.00
yes	rs12916	5	75360714	HMGCR	T/C	38.0	0.56	6.4	42.2	0.557	0.30
yes	rs1532624	16	56971567	CETP	C/A	41.5	0.71	6.4	40.8	0.634	0.70
yes	rs1695	11	67585218	GSTP1	A/G	31.6	1.00	6.4	27.8	1.000	0.00
yes	rs17222723	10	99836239	ABCC2	T/A	13.0	0.69	8.0	12.6	1.000	0.00
yes	rs17238484	5	75352671	HMGCR	G/T	22.6	0.43	8.0	25.8	0.548	0.70
yes	rs17238540	5	74655498	HMGCR	T/G	6.0	0.34	7.2	4.0	0.008	0.30
MONOMORPHIC	rs1799837	11	116837537	APOA1	C	0.0	-	6.4	0.7	1.000	0.00
yes	rs1799853	10	94942290	CYP2C9	C/T	12.8	0.69	6.4	16.9	0.410	0.00
yes	rs1800629	6	31575254	TNF	G/A	10.3	1.00	6.4	8.6	0.146	0.00
yes	rs1800795	7	22727026	IL6	G/C	31.9	0.39	7.2	28.2	0.887	0.30
yes	rs1800896	1	206773552	IL10	T/C	43.2	1.00	6.4	37.3	0.325	0.00
yes	rs2010963	6	43770613	VEGFA	G/C	37.6	0.56	6.4	38.4	0.904	0.00
yes	rs2228314	22	41880738	SREBF2	G/C	24.6	0.45	7.2	26.3	0.374	0.00
MAF	rs2740574	7	99784473	CYP3A4	T/C	1.7	1.00	6.4	1.7	1.000	0.00
yes	rs3025039	6	43784799	VEGFA	C/T	14.1	1.00	6.4	14.7	0.492	0.00
yes	rs328	8	19962213	LPL	C/G	12.4	0.69	6.4	12.7	0.799	0.00
yes	rs35599367	7	99366316	CYP3A4	G/A	6.4	1.00	6.4	5.1	0.556	0.00
MONOMORPHIC	rs366631	1	109709850	GSTM5	A	0.0	-	6.4	0.0	NA	0.00
yes	rs3746444	20	34990448	MYH7B	A/G	24.1	0.21	7.2	28.5	0.324	0.00
yes	rs3761740	5	75336308	HMGCR	C/A	8.8	0.60	9.6	8.9	0.718	0.00
yes	rs4149056	12	21331549	SLCO1B1	T/C	14.8	1.00	8.0	12.6	0.194	0.00
yes	rs4363657	12	21368722	SLCO1B1	T/C	15.4	1.00	6.4	13.4	0.801	1.00
yes	rs4636297	9	136670698	EGFL7	G/A	39.3	0.13	6.4	45.7	0.644	0.00
yes	rs4693570	4	83170698	100 kb downstream of COQ2	C/T	40.9	0.34	7.2	41.4	0.235	0.00
yes	rs4880	6	159692840	SOD2	A/G	46.6	1.00	6.4	49.0	0.645	0.00
yes	rs662	7	95308134	PON1	T/C	33.8	1.00	6.4	27.2	0.884	0.00
yes	rs699947	6	43768652	VEGFA	C/A	38.5	0.85	6.4	44.5	0.907	0.00
yes	rs705379	7	95324583	PON1	A/G	46.5	1.00	9.6	46.8	0.353	1.00
yes	rs708272	16	56962376	CETP	G/A	40.0	1.00	8.0	39.1	0.717	0.00
yes	rs717620	10	99782821	ABCC2	C/T	17.5	0.75	6.4	16.6	0.411	0.00
MONOMORPHIC	rs72558195	10	95064886	CYP2C8	G	0.0	-	6.4	0.2	1.000	0.00
yes	rs776746	7	99672916	CYP3A5	C/T	4.7	1.00	6.4	5.6	0.610	0.00
yes	rs8187710	10	99851537	ABCC2	G/A	13.2	0.69	6.4	12.3	1.000	0.00

Supplementary Table S2. Effects of the genotype of the SNPs significantly associated with baseline lipid traits in the treatment-naïve cohort on the response to 12-week phytochemical treatment.

			p-values					
SNP	Gene	df	TC	LDL-C	HDL-C	TRIG	TC:HDL-C ratio	non-HDL-C
rs1532624	CETP	2, 109	0.165	0.147	0.233	0.513	0.481	0.163
rs1800629	TNFA	2, 112	0.569	0.383	0.987	0.208	0.770	0.504
rs3746444	MYH7B miR-499	2, 111	0.560	0.305	0.459	0.873	0.249	0.350
rs4880	SOD2	2, 109	0.584	0.497	0.320	0.581	0.657	0.786
rs699947	VEGFA	2, 109	0.154	0.391	0.976	0.466	0.754	0.092
rs708272	CETP	2, 107	0.312	0.089	0.264	0.713	0.628	0.352

P-values for repeated measures ANOVA. Models adjusted for age and gender. df, degrees of freedom.

Supplementary Table S3. Summary of the predicted miR-499a-5p target genes belonging to pathways related to lipid and lipoprotein metabolism, resulting from the intersection between the TargetScan and Pathcards databases.

Metabolism Superpath, which includes…

Metabolism of lipids and lipoproteins pathway, which includes…	Lipoprotein metabolism Superpath, which includes…	11 Pathways in the Lipoprotein metabolism SuperPath										
		VLDL biosynthesis	Digestion of dietary lipid	Lipid digestion, mobilization, and transport	Chylomicron-mediated lipid transport	Hormone-sensitive lipase (HSL)-mediated triacylglycerol hydrolysis	Reelin signalling pathway	HDL-mediated lipid transport	VLDL interactions	LDL-mediated lipid transport	VLDLR internalisation and degradation	Lipoprotein metabolism
ACBD5												
AHR												
AKR1D1												
ARSJ												
CDK19												
CHD9												
CROT												
FDX1												
FYN	FYN			FYN			FYN		FYN			FYN
INPP5E												
LBR												
LIPA	LIPA			LIPA						LIPA		LIPA
MED13												
MED7												
NPC1L1	NPC1L1			NPC1L1								
OSBPL1A												
PRKAA2												
SH3KBP1	SH3KBP1			SH3KBP1			SH3KBP1		SH3KBP1			SH3KBP1
SLC44A1												
STS												
TNFAIP8L3												
UGCG												
VAPA												
